# Effect of semaglutide on major adverse cardiovascular events by baseline kidney parameters in participants with type 2 diabetes and at high risk of cardiovascular disease: SUSTAIN 6 and PIONEER 6 *post hoc* pooled analysis

**DOI:** 10.1186/s12933-023-01949-7

**Published:** 2023-08-24

**Authors:** Peter Rossing, Stephen C. Bain, Heidrun Bosch-Traberg, Ekaterina Sokareva, Hiddo J. L. Heerspink, Søren Rasmussen, Linda G. Mellbin

**Affiliations:** 1 Steno Diabetes Center, Copenhagen, Denmark; 2 Swansea University Medical School, Swansea, UK; 3 Novo Nordisk A/S, Søborg, Denmark; 4 University of Groningen, Groningen, the Netherlands; 5 Karolinska Institutet, Solna, Sweden

**Keywords:** Type 2 diabetes, Cardiovascular disease, Kidney disease, Glucagon-like peptide-1 receptor agonist, Semaglutide, Major cardiovascular events, Estimated glomerular filtration rate, Urine albumin:creatinine ratio, NCT01720446, NCT02692716

## Abstract

**Background:**

Semaglutide is a glucose-lowering treatment for type 2 diabetes (T2D) with demonstrated cardiovascular benefits; semaglutide may also have kidney-protective effects. This *post hoc* analysis investigated the association between major adverse cardiovascular events (MACE) and baseline kidney parameters and whether the effect of semaglutide on MACE risk was impacted by baseline kidney parameters in people with T2D at high cardiovascular risk.

**Methods:**

Participants from the SUSTAIN 6 and PIONEER 6 trials, receiving semaglutide or placebo, were categorised according to baseline kidney function (estimated glomerular filtration rate [eGFR] < 45 and ≥ 45–<60 versus ≥ 60 mL/min/1.73 m^2^) or damage (urine albumin:creatinine ratio [UACR] ≥ 30–≤300 and > 300 versus < 30 mg/g). Relative risk of first MACE by baseline kidney parameters was evaluated using a Cox proportional hazards model. The same model, adjusted with inverse probability weighting, and a quadratic spline regression were applied to evaluate the effect of semaglutide on risk and event rate of first MACE across subgroups. The semaglutide effects on glycated haemoglobin (HbA_1c_), body weight (BW) and serious adverse events (SAEs) across subgroups were also evaluated.

**Results:**

Independently of treatment, participants with reduced kidney function (eGFR ≥ 45–<60 and < 45 mL/min/1.73 m^2^: hazard ratio [95% confidence interval]; 1.36 [1.04;1.76] and 1.52 [1.15;1.99]) and increased albuminuria (UACR ≥ 30–≤300 and > 300 mg/g: 1.53 [1.14;2.04] and 2.52 [1.84;3.42]) had an increased MACE risk versus those without. Semaglutide consistently reduced MACE risk versus placebo across all eGFR and UACR subgroups (interaction p value [p_INT_] > 0.05). Semaglutide reduced HbA_1c_ regardless of baseline eGFR and UACR (p_INT_>0.05); reductions in BW were affected by baseline eGFR (p_INT_<0.001) but not UACR (p_INT_>0.05). More participants in the lower eGFR or higher UACR subgroups experienced SAEs versus participants in reference groups; the number of SAEs was similar between semaglutide and placebo arms in each subgroup.

**Conclusions:**

MACE risk was greater for participants with kidney impairment or damage than for those without. Semaglutide consistently reduced MACE risk across eGFR and UACR subgroups, indicating that semaglutide provides cardiovascular benefits in people with T2D and at high cardiovascular risk across a broad spectrum of kidney function and damage.

**Trial registrations:**

NCT01720446; NCT02692716.

**Supplementary Information:**

The online version contains supplementary material available at 10.1186/s12933-023-01949-7.

## Background

Glucagon-like peptide-1 receptor agonists (GLP-1RAs), including once-weekly (OW) subcutaneous (s.c.) and once-daily (OD) oral formulations of semaglutide, are a well-established treatment option for type 2 diabetes (T2D), with proven beneficial effects on glycated haemoglobin (HbA_1c_) and body weight (BW) [1]. Cardiovascular outcomes trials (CVOTs) have shown that several GLP-1RAs, including semaglutide, reduced the relative risk of major adverse cardiovascular events (MACE) versus placebo in people with T2D and cardiovascular disease (CVD) or at high cardiovascular (CV) risk (non-inferiority for the risk of MACE with the OD oral formulation of semaglutide), without additional safety concerns [2–6]. In a meta-analysis evaluating CVOTs, it was shown that GLP-1RA treatment reduced MACE risk by 14% versus placebo (p < 0.0001) in participants with T2D and with, or at high/very high risk of, CVD [7].

Based on data from clinical trials in the field of GLP-1RAs and sodium–glucose cotransporter-2 inhibitors (SGLT2is), current guidance from the American Diabetes Association (ADA) and European Association for the Study of Diabetes (EASD), American Heart Association and European Society of Cardiology recommend GLP-1RAs and SGLT2is as first-line therapies for glycaemic, weight and CVD management in people with T2D and with, or at high/very high risk of, atherosclerotic CVD [8–10]. Notably, recent treatment guidance from the ADA and EASD consensus statement and Kidney Disease: Improving Global Outcomes (KDIGO) guidelines state that GLP-1RAs with proven CV benefits can be used as second-line glucose-lowering therapy for people with T2D and chronic kidney disease (CKD) specifically, who have not achieved their glycaemic target with metformin and/or SGLT2is or when SGLT2is are contraindicated [10–12].

In contrast to the established kidney-related effects of SGLT2is from kidney outcomes trials [13], there are currently no data from kidney outcomes trials demonstrating efficacy of GLP-1RAs regarding kidney-related endpoints [16]. However, several CVOTs have included participants with reduced kidney function [2–5, 14], enabling the introduction of GLP-1RAs in guidelines and consensus statements addressing CKD treatment [10–12]. A meta-analysis including data from GLP-1RA CVOTs has shown that GLP-1RAs reduced the risk of MACE versus placebo in people with T2D at high CV risk, including in those with low baseline estimated glomerular filtration rate (eGFR) [7]. Moreover, GLP-1RAs reduced the relative risk for a composite kidney outcome (time to development of macroalbuminuria, doubling of serum creatinine or ≥ 40% eGFR decline, kidney replacement therapy, or kidney-related death) compared with placebo [7]; an earlier CVOT meta-analysis indicated that this effect of GLP-1RAs was largely driven by a reduction in albuminuria [15]. In participants with T2D and CKD (stages 3–4), the GLP-1RA dulaglutide reduced decline in eGFR as a secondary outcome [16].

Previous *post hoc* analyses of the SUSTAIN and PIONEER programmes have evaluated the effect of semaglutide in people with T2D and established CVD and/or CKD, or CV risk factors [17–20], and how CKD status affects semaglutide treatment outcomes [17, 19, 20]. Semaglutide consistently lowered HbA_1c_ versus placebo across eGFR levels, whereas weight loss was greater in participants with baseline eGFR < 60 mL/min/ 1.73 m^2^ than in those with baseline eGFR ≥ 60 mL/min/1.73 m^2^ (SUSTAIN 6 and 10 and PIONEER 5 and 6) [17]. Moreover, semaglutide reduced the annual rate of eGFR decline versus placebo, regardless of baseline eGFR and urine albumin:creatinine ratio (UACR) [19, 20]. Although a previous *post hoc* analysis based on SUSTAIN 6 and PIONEER 6 (N = 6,480) showed that semaglutide reduced the risk of MACE versus placebo, it was not interrogated how CKD status may affect this outcome [18]. Consequently, it is not fully understood how eGFR and UACR at baseline affect the risk of MACE in T2D, and if there is an interaction between these kidney parameters and semaglutide treatment outcomes. The aim of this *post hoc* analysis was to evaluate (1) the association between MACE and baseline kidney parameters, namely kidney function (eGFR) or damage (UACR), and (2) the effect of semaglutide on risk of MACE by baseline kidney parameters, in a pooled population from SUSTAIN 6 and PIONEER 6, comprising of people with T2D and at high CV risk.

## Methods

### Trial designs and participants

Participants from SUSTAIN 6 and PIONEER 6 who received OW s.c. semaglutide 0.5 or 1.0 mg or OD oral semaglutide 14 mg, respectively, or volume-matched placebo for 2.1 years and 15.9 months, respectively, were pooled for this *post hoc* analysis. The study designs for SUSTAIN 6 (NCT01720446) and PIONEER 6 (NCT02692716) have been reported in detail elsewhere; however, specific details are included here for clarity [5, 6].

Key inclusion criteria for participants in the SUSTAIN 6 and PIONEER 6 CVOTs were age of ≥ 50 years and established CVD (including previous CV, cerebrovascular or peripheral vascular disease), chronic heart failure or CKD, or age of ≥ 60 years and at least one CV risk factor [5, 6]. In both CVOTs, key kidney-related exclusion criteria were long-term or intermittent haemodialysis or peritoneal dialysis [5, 6]; for PIONEER 6, severe kidney impairment classified as GFR < 30 mL/min/1.73 m^2^ was also an exclusion criterion [6].

### eGFR and UACR subgroups

The current study population was subdivided according to baseline eGFR and UACR based on categories in the 2022 KDIGO guidelines [12]. eGFR subgroups comprised data from both SUSTAIN 6 and PIONEER 6 (n = 6,461). To evaluate MACE risk by kidney function, participants were divided into the following groups: ≥60 mL/min/1.73 m^2^ (CKD stage 1 or 2), ≥ 45–<60 mL/min/1.73 m^2^ (CKD stage 3a) and < 45 mL/min/1.73 m^2^ (CKD stage 3b or ≥ 4; including participants with < 30 mL/min/1.73 m^2^ from SUSTAIN 6) based on the CKD-Epidemiology Collaboration equation [21]. To assess kidney damage, UACR subgroups were evaluated, comprising data from SUSTAIN 6 only (n = 3,238), as this parameter was not assessed in PIONEER 6 [6]. Participants were divided into the following groups: <30 mg/g (normal UACR), ≥ 30–≤300 mg/g (moderately increased UACR) and > 300 mg/g (severely increased UACR).

### Safety profile of semaglutide across baseline eGFR and UACR subgroups

The number and proportion of participants with more than one serious adverse event (SAE) were reported by baseline eGFR and UACR subgroups, in addition to per treatment arm within each kidney parameter subgroup. SAEs were coded according to the Medical Dictionary for Regulatory Activities version 18.0 and 20.1 in the SUSTAIN 6 and PIONEER 6 CVOTs, respectively.

### Statistical analyses

Descriptive baseline characteristics were based on the full analysis set, including all randomised participants with eGFR and UACR values at baseline. All statistical analyses were based on the full analysis set using in-trial data. In-trial data were defined as information collected at or after the randomisation date until end-of-trial follow-up visit, death of participant or withdrawal of consent.

MACE was defined as a composite of CV death, non-fatal myocardial infarction and non-fatal stroke. The subgroup and treatment effects on time to first event of MACE by baseline kidney function and albuminuria status were analysed with a stratified Cox proportional hazards model; data are presented as hazard ratio (HR) and 95% confidence intervals (CI). For the evaluation of MACE risk by baseline eGFR and UACR, treatment (semaglutide, placebo) and subgroup were used as explanatory variables in the model. Treatment, subgroup, and the interaction of treatment and subgroup were used as explanatory variables when assessing the effect of semaglutide on MACE risk across kidney parameter subgroups. The Cox proportional hazards model was stratified by trial and CV risk group (established CVD versus risk factors) for the eGFR subgroup analyses, as data from the SUSTAIN 6 and PIONEER 6 CVOTs were pooled. For the UACR subgroup analyses, the model was stratified by CV risk group only because UACR was not measured in PIONEER 6 [6]. Because the model was applied to evaluate the risk of first MACE in participants with different eGFR and UACR values, the subgroups eGFR ≥ 60 mL/min/1.73 m^2^ (normal or mildly reduced kidney function) and UACR < 30 mg/g (normoalbuminuria) were used as references.

The main analyses in this study were with adjusted Cox proportional hazards models. For the evaluation of MACE risk by baseline eGFR and UACR, regardless of treatment, the Cox proportional hazards model was adjusted for the following baseline predictors of cardiorenal disease: age, gender, diabetes duration, glucose-lowering agent (yes/no), smoking status, HbA_1c_, previous myocardial infarction/stroke/transient ischaemic attack, and geographic region. The effect of semaglutide on MACE risk across kidney parameter subgroups was assessed by applying the Cox proportional hazards model with adjustment using inverse probability weighting based on a logistic regression model. Treatment was used as dependent variable, and the adjusted model included the abovementioned baseline predictors of cardiorenal disease in addition to continuous eGFR or UACR values at baseline.

The absolute risk reduction (ARR) for the first MACE was calculated for each kidney parameter subgroup, using a method previously published by Altman and Kragh Andersen [22].

A quadratic spline in the Cox proportional hazards model was applied to calculate predicted event rate of first MACE by 2 years across a continuum of baseline eGFR and UACR values, both in pooled treatment arms and per treatment arm. The event rate is defined as the probability (number of individuals per 100 individuals) to experience a first MACE within 2 years. For the UACR continuum, baseline values were transformed into natural logarithmic (*ln*) values.

To investigate if baseline kidney function and albuminuria status affect the effect of semaglutide on glycaemic control and weight loss, HbA_1c_ and BW were evaluated in the defined eGFR and UACR subgroups. Post-baseline responses were analysed using a mixed model for repeated measurements with treatment by subgroup as a fixed factor; adjustment for baseline HbA_1c_ or BW and trial (SUSTAIN 6 and PIONEER 6) was applied. For the eGFR subgroup analysis, visits that occurred at different weeks in SUSTAIN 6 and PIONEER 6 were combined; treatment effects by subgroups were evaluated at combined weeks 80 (SUSTAIN 6) and 83 (PIONEER 6).

Tests for heterogeneity in treatment effect across eGFR and UACR subgroups were indicated by interaction p values (p_INT_), with p < 0.05 indicating a significant interaction. No adjustment for multiplicity was performed.

## Results

### Baseline characteristics

Baseline characteristics, including medications and kidney-related parameters, for the current study population are shown in Table 1.

**Table 1:** Baseline characteristics by baseline kidney function

	eGFR subgroup (mL/min/1.73 m^2^); N = 6,461			UACR subgroup (mg/g)*; N = 3,238		
	≥ 60	≥ 45–<60	< 45	< 30	≥ 30–≤300	> 300
Number of participants	4,762	968	731	1,934	884	420
Age, years	64.3 ± 6.9	68.2 ± 7.1	68.8 ± 7.8	64.5 ± 7.4	65.5 ± 7.3	63.5 ± 7.1
Sex, male	3,133 (65.8)	611 (63.1)	422 (57.7)	1,125 (58.2)	585 (66.2)	256 (61.0)
Body weight, kg	91.1 ± 20.6	94.1 ± 21.8	91.1 ± 21.6	92.6 ± 19.8	91.9 ± 21.2	89.9 ± 22.6
T2D duration, years	13.6 ± 8.0	15.9 ± 8.8	17.6 ± 8.6	12.9 ± 8.0	14.5 ± 7.8	17.0 ± 8.3
HbA_1c_, %	8.5 ± 1.6	8.3 ± 1.5	8.3 ± 1.5	8.5 ± 1.4	8.9 ± 1.5	9.2 ± 1.6
HbA_1c_, mmol/mol	69.2 ± 17.2	67.4 ± 16.9	67.3 ± 16.1	69.7 ± 14.8	73.2 ± 16.6	76.6 ± 18.0
SBP, mmHg	135.4 ± 17.0	135.5 ± 17.6	137.3 ± 19.4	133.1 ± 15.9	136.4 ± 16.5	145.7 ± 19.6
DBP, mmHg	77.0 ± 9.8	75.4 ± 10.3	75.3 ± 10.7	76.7 ± 9.8	76.5 ± 9.9	79.9 ± 10.9
Prior CV event	2,231 (46.9)	372 (38.4)	271 (37.1)	906 (46.8)	353 (39.9)	164 (39.0)
Total cholesterol, mmol/L	4.3 ± 1.1	4.3 ± 1.2	4.4 ± 1.3	4.4 ± 1.2	4.4 ± 1.1	4.8 ± 1.5
HDL cholesterol, mmol/L	1.1 ± 0.3	1.1 ± 0.3	1.1 ± 0.3	1.2 ± 0.3	1.2 ± 0.3	1.2 ± 0.4
LDL cholesterol, mmol/L	2.3 ± 0.9	2.2 ± 0.9	2.3 ± 1.0	2.3 ± 0.9	2.3 ± 0.9	2.6 ± 1.2
Triglycerides, mmol/L	2.0 ± 1.5	2.2 ± 1.7	2.2 ± 1.6	2.0 ± 1.7	2.2 ± 1.5	2.4 ± 1.6
**Glucose-lowering medication**						
Metformin	3,986 (83.7)	622 (64.3)	258 (35.3)	1,518 (78.5)	629 (71.2)	226 (53.8)
Sulphonylurea	1,853 (38.9)	362 (37.4)	213 (29.1)	878 (45.4)	354 (40.0)	156 (37.1)
Thiazolidinedione	132 (2.8)	29 (3.0)	33 (4.5)	34 (1.8)	18 (2.0)	19 (4.5)
Glinides	99 (2.1)	17 (1.8)	21 (2.9)	51 (2.6)	24 (2.7)	11 (2.6)
Insulins	2,381 (50.0)	590 (61.0)	502 (68.7)	815 (42.1)	468 (52.9)	236 (56.2)
Other^†^	364 (7.6)	54 (5.6)	21 (2.9)	29 (1.5)	13 (1.5)	9 (2.1)
**CV medication at baseline**						
Beta blockers	2,787 (58.5)	577 (59.6)	409 (56.0)	1,129 (58.4)	479 (54.2)	244 (58.1)
Calcium channel blockers	1,400 (29.4)	347 (35.8)	331 (45.3)	501 (25.9)	333 (37.7)	206 (49.0)
ACE inhibitors	2,270 (47.7)	422 (43.6)	282 (38.6)	996 (51.5)	446 (50.5)	171 (40.7)
ARB	1,638 (34.4)	407 (42.0)	320 (43.8)	589 (30.5)	311 (35.2)	190 (45.2)
Diuretics	1,607 (33.7)	473 (48.9)	437 (59.8)	706 (36.5)	340 (38.5)	188 (44.8)
Lipid-lowering drugs	3,777 (79.3)	795 (82.1)	618 (84.5)	1,485 (76.8)	667 (75.5)	322 (76.7)
Platelet aggregation inhibitors^‡^	3,614 (75.9)	675 (69.7)	489 (66.9)	1,463 (75.6)	629 (71.2)	276 (65.7)
Anti-thrombotic medication^§^	277 (5.8)	98 (10.1)	65 (8.9)	111 (5.7)	58 (6.6)	19 (4.5)
**Kidney parameters**						
eGFR, mL/min/1.73 m^2^	85.7 ± 13.7	52.4 ± 4.3	35.6 ± 7.4	80.5 ± 20.0	73.6 ± 23.0	58.5 ± 25.9
eGFR, mL/min/1.73 m^2^ ≥ 90 ≥ 60–<90 ≥ 30–<60 < 30						
≥ 90	2,053 (43.1)	–	–	768 (39.7)	273 (30.9)	70 (16.7)
≥ 60–<90	2,709 (56.9)	–	–	828 (42.8)	344 (38.9)	125 (29.8)
≥ 30–<60	–	968 (100.0)	592 (81.0)	316 (16.3)	247 (27.9)	157 (37.4)
< 30	–	–	139 (19.0)	22 (1.1)	20 (2.3)	68 (16.2)
Participants with UACR values*	2,408 (50.6)	436 (45.0)	394 (53.9)	1,934 (100.0)	884 (100.0)	420 (100.0)
UACR, mg/g, geometric mean (%CV)*	17.4 (487.9)	34.6 (824.4)	121.8 (1,507)	6.1 (113.4)	83.6 (72.1)	990.5 (102.0)
UACR, mg/g*						
< 30	1,596 (66.3)	214 (49.1)	124 (31.5)	1,934 (100.0)	–	–
≥ 30–≤300	617 (25.6)	148 (33.9)	119 (30.2)	–	884 (100.0)	–
> 300	195 (8.1)	74 (17.0)	151 (38.3)	–	–	420 (100.0)

*SUSTAIN 6 data only; UACR was not measured in PIONEER 6. ^†^The category ‘Other’ includes alpha glucosidase inhibitors, DPP-4is and SGLT2is. ^‡^The category ‘Platelet aggregation inhibitors’ comprised acetylsalicylic acid or adenosine diphosphate receptor inhibitors (excluding acetylsalicylic acid). ^§^The category ‘Anti-thrombotic medication’ comprised vitamin K antagonists, direct thrombin inhibitors or direct factor Xa inhibitors

Data are presented as mean ± SD or numbers and proportions (%) of participants. Prior CV event is defined as prior myocardial infarction, stroke or transient ischaemic attack

%CV, coefficient of variation; ACE, angiotensin-converting enzyme; ARB, angiotensin receptor blocker; CV, cardiovascular; DBP, diastolic blood pressure; DDP-4i, dipeptidyl peptidase-4 inhibitor; eGFR, estimated glomerular filtration rate; HDL, high-density lipoprotein; LDL, low-density lipoprotein; N, total number of participants included in eGFR or UACR subgroup analyses; n, number of participants; SBP, systolic blood pressure; SD, standard deviation; SGLT2i, sodium–glucose cotransporter-2 inhibitor; UACR, urine albumin:creatinine ratio

At baseline, most participants had normal or mildly reduced kidney function and were normoalbuminuric. The baseline characteristics of the study population were generally balanced across eGFR and UACR subgroups; the mean age ranged between 63.5 and 68.8 years, most participants were males (57.7–66.2%), and the participants had longstanding T2D (mean duration 12.9–17.6 years). The most frequently used glucose-lowering agents in the current study population were metformin, insulins and sulphonylurea. Across all subgroups, the CVD medications that most participants were treated with were: lipid-lowering drugs (75.5–84.5%), platelet aggregation inhibitors (65.7–75.9%) and beta blockers (54.2–59.6%).

At baseline, 139 (19.0%), 22 (1.1%), 20 (2.3%) and 68 (16.2%) participants in the eGFR < 45 mL/min/1.73 m^2^, UACR < 30 mg/g, UACR ≥ 30–≤300 mg/g and UACR > 300 mg/g subgroups, respectively, had an eGFR < 30 mL/min/1.73 m^2^.

### Risk of MACE by baseline kidney function and albuminuria status

**Fig. 1:**
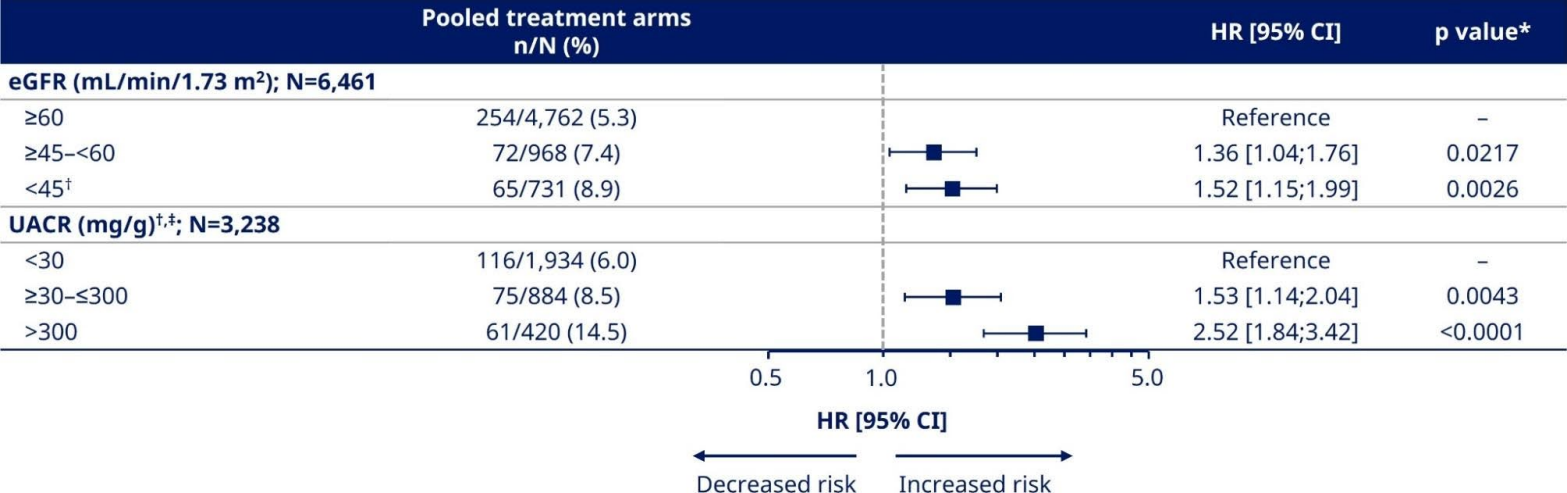
Unadjusted analysis for risk of MACE by baseline eGFR and UACR regardless of treatment. *p values indicated for comparisons with reference groups (eGFR ≥ 60 mL/min/1.73 m^2^ and UACR < 30 mg/g). ^†^Participants with eGFR < 30 mL/min/1.73 m^2^ from SUSTAIN 6 were included for the analysis in the eGFR < 45 mL/min/1.73 m^2^ as well as UACR < 30, ≥30–≤300, and > 300 mg/g subgroups with the following numbers (%): 139 (19.0); 22 (1.1); 20 (2.3); 68 (16.2). ^‡^SUSTAIN 6 data only; UACR was not measured in PIONEER 6. %, proportion of participants with ≥ 1 cardiovascular event; CI, confidence interval; eGFR, estimated glomerular filtration rate; HR, hazard ratio; MACE, major adverse cardiovascular event; N, total number of participants in pooled population or subgroup with eGFR or UACR values at baseline; n, number of participants with ≥ 1 cardiovascular event; UACR, urine albumin:creatinine ratio

When assessing the risk of MACE across eGFR subgroups, regardless of treatment the risk of MACE was higher in participants with eGFR ≥ 45–<60 mL/min/1.73 m^2^ and < 45 mL/min/1.73 m^2^ (HR [95% CI] 1.36 [1.04;1.76] and 1.52 [1.15;1.99], respectively), compared with the eGFR ≥ 60 mL/min/1.73 m^2^ subgroup (p < 0.05 for all comparisons; Fig. 1 and Supplementary Table 1). The risk for first MACE increased in all eGFR subgroups after adjustment for baseline variables (HR [95% CI] ≥ 45–<60 mL/min/1.73 m^2^: 1.50 [1.13;1.96] and < 45 mL/min/1.73 m^2^: 1.71 [1.27;2.28]; p < 0.05 versus ≥ 60 mL/min/1.73 m^2^ for all comparisons; Supplementary Table 1).

For the UACR subgroups, there was an increase in the risk of MACE with increased albuminuria regardless of treatment. The HR [95% CI] for participants with UACR ≥ 30–≤300 and > 300 mg/g were 1.53 [1.14;2.04] and 2.52 [1.84;3.42] compared with the UACR < 30 mg/g subgroup (p < 0.05 for all comparisons; Fig. 1 and Supplementary Table 1). There was an attenuation of MACE risk in all UACR subgroups after adjustment for baseline variables (HR [95% CI] ≥ 30–≤300 mg/g: 1.43 [1.06;1.91] and > 300 mg/g: 2.37 [1.70;3.27]; p < 0.05 versus < 30 mg/g for all comparisons; Supplementary Table 1).

### Effect of semaglutide on the relative risk and absolute risk reduction of MACE by baseline kidney function and albuminuria status

**Fig. 2:**
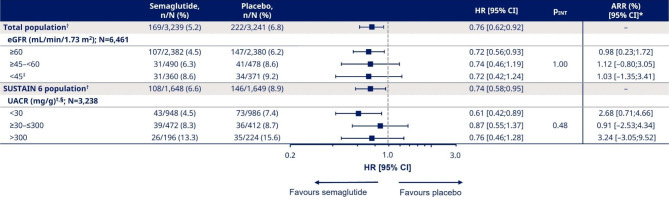
Adjusted analysis for the effect of semaglutide on MACE risk by baseline eGFR and UACR. *Cut-off point for ARR was 1 year for eGFR and 2 years for UACR. ^†^Data previously published in Husain [18] (N numbers for total randomised participants, regardless of available baseline eGFR or UACR values, were 6,480 [total] and 3,297 [SUSTAIN 6]). ^‡^Participants with eGFR < 30 mL/min/1.73 m^2^ from SUSTAIN 6 were included for the analysis in the eGFR < 45 mL/min/1.73 m^2^ as well as UACR < 30, ≥30–≤300, and > 300 mg/g subgroups with the following numbers (%): 139 (19.0); 22 (1.1); 20 (2.3); 68 (16.2). ^§^SUSTAIN 6 data only; UACR was not measured in PIONEER 6. A Cox proportional hazards model was adjusted with inverse probability weighting, using baseline predictors of cardiorenal disease and continuous eGFR or UACR values at baseline. %, proportion of participants with ≥ 1 cardiovascular event; ARR, absolute risk reduction; CI, confidence interval; eGFR, estimated glomerular filtration rate; HR, hazard ratio; MACE, major adverse cardiovascular event; N, total number of participants in pooled population or subgroup with eGFR or UACR values at baseline; n, number of participants with ≥ 1 cardiovascular event; p_INT_, interaction p value; UACR, urine albumin:creatinine ratio

As previously demonstrated in a *post hoc* analysis of the pooled SUSTAIN 6 and PIONEER 6 population, the HR for the effect on overall MACE risk was 0.76 [95% CI 0.62;0.92] in favour of semaglutide versus placebo [18] (Fig. 2).

The current analysis of the impact of baseline kidney parameters on the effect of semaglutide on MACE risk showed consistent reductions in the relative risk versus placebo across all eGFR and UACR subgroups after adjustment for baseline variables.

The treatment effects for semaglutide versus placebo for first MACE were similar across eGFR subgroups (Fig. 2); HRs [95% CI] for participants with eGFR ≥ 60, ≥45–<60 and < 45 mL/min/1.73 m^2^ were 0.72 [0.56;0.93], 0.74 [0.46;1.19] and 0.72 [0.42;1.24], respectively (p_INT_ = 1.00; Fig. 2). The ARRs for participants with eGFR ≥ 60, ≥45–<60 and < 45 mL/min/1.73 m^2^ were 0.98%, 1.12% and 1.03%, respectively.

Correspondingly, for the UACR subgroups, the HRs [95% CI] for participants with UACR < 30, ≥30–≤300 and > 300 mg/g were 0.61 [0.42;0.89], 0.87 [0.55;1.37] and 0.76 [0.46;1.28], respectively (Fig. 2). The p_INT_ value indicated that there was no treatment heterogeneity across UACR subgroups (p_INT_ = 0.48). The ARRs for participants with UACR < 30, ≥30–≤300 and > 300 mg/g were 2.68%, 0.91% and 3.24%, respectively.

Observations from the unadjusted analysis were in line with these results (Supplementary Table 2).

### Event rate of first MACE by 2 years across continuums of baseline eGFR and UACR values

**Fig. 3:**
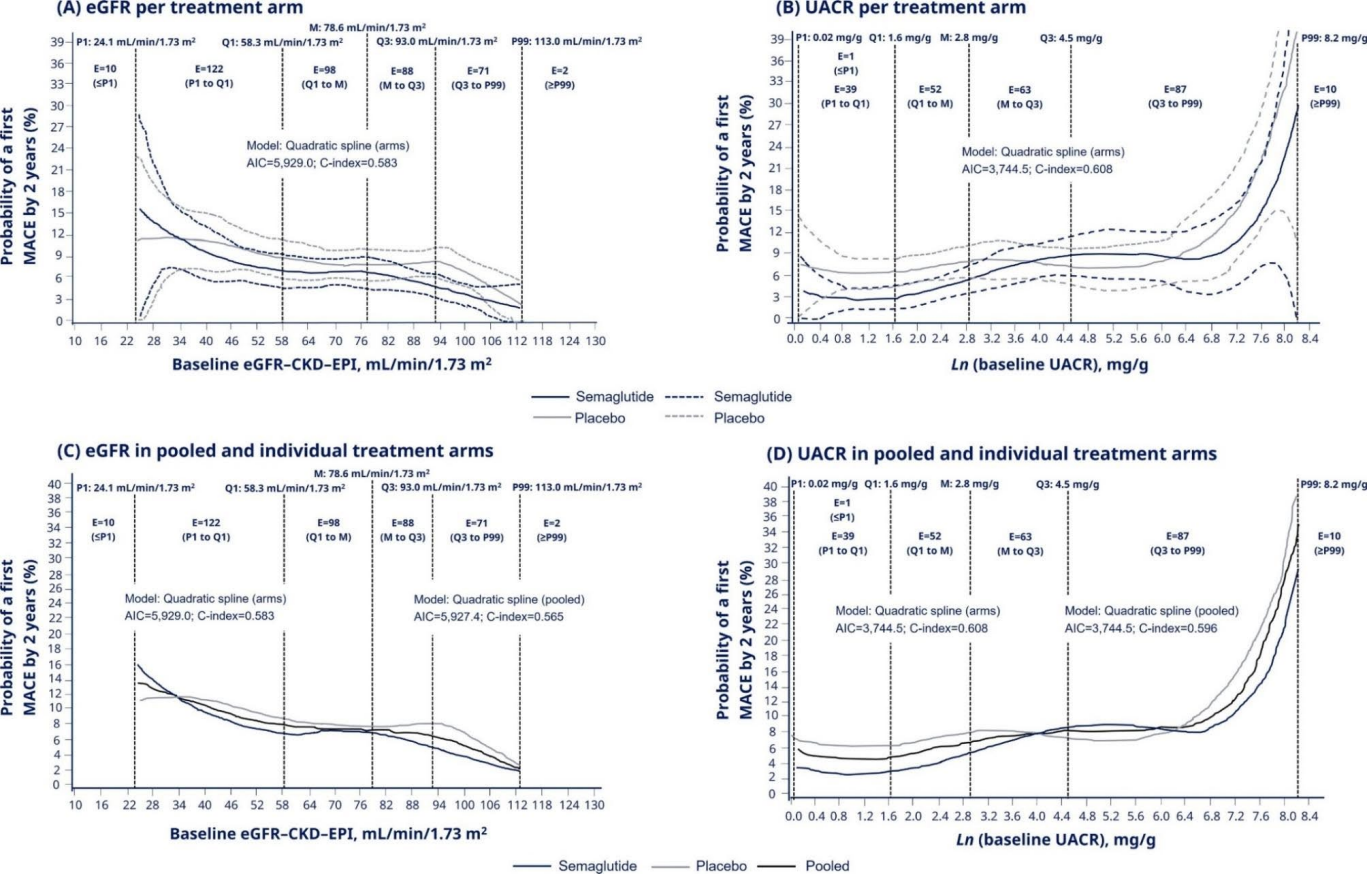
Event rate of first MACE by 2 years* by baseline eGFR and UACR values. *The event rate is defined as the probability (number of individuals per 100 individuals) to experience a first MACE within 2 years. A quadratic spline regression, using the Cox proportional hazard model, on predicted event rate of MACE by 2 years was calculated in per treatment arm across **(A)** eGFR and **(B)** UACR values, as well as in pooled treatment arms across the continuum of **(C)** eGFR and **(D)** UACR values. Data are presented as medians (solid lines); vertical black dashed lines represent percentile, quartile and median values. In panels A and B, blue and grey dashed lines represent 95% CIs for semaglutide and placebo, respectively. Baseline UACR values are transformed to the natural logarithmic (*Ln*) values. The number of events in defined intervals for each kidney parameter continuum are presented within the vertical black dashed lines defining the interval (two events occurred in participants without available UACR data at baseline). AIC, Akaike information criterion; CI, confidence interval; C-index, concordance index; CKD-EPI, Chronic Kidney Disease Epidemiology Collaboration; eGFR, estimated glomerular filtration rate; E, number of events; M, median; MACE, major adverse cardiovascular event; P, percentile; Q, quartile; UACR, urine albumin:creatinine ratio

The event rate of first MACE by 2 years was estimated across a continuum of eGFR (from first percentile [P1] to 99th percentile [P99]: 24.1–113.0 mL/min/1.73 m^2^) and UACR (from P1 to P99: 1.02–3,713.6 mg/g; presented as *ln* values) values (Fig. 3A–D).

In line with the subgroup analyses, the MACE event rate was lower in the semaglutide arm than in the placebo arm across the continuum of eGFR values (number of events: at and below P1: 10; P1 to first quartile [Q1]: 122; Q1 to median: 98; median to third quartile [Q3]: 88; Q3 to P99: 71; at or above the P99: 2), except at the lower end of the eGFR continuum as events became sparse (as indicated by low number of events below and at P1 and wide 95% CIs; Fig. 3A). There were variations in the event rate of MACE between the semaglutide and placebo arms across the continuum of UACR values; however, there was a small number of events in several intervals of the continuum (number of events: at and below the UACR P1: 1; P1 to Q1: 39; Q1 to median: 52; median to Q3: 63; Q3 to P99: 87; at or above the P99: 10) (Fig. 3B).

When analysing the event rate of first MACE in pooled treatment arms (semaglutide and placebo) in the continuum of eGFR and UACR, MACE risk was higher with lower eGFR values and higher UACR values (Fig. 3C, D) in line with findings from the subgroup analyses (Fig. 1 and Supplementary Table 1).

### Effect of semaglutide on HbA_1c_ and weight loss by baseline kidney function and albuminuria status

Semaglutide consistently reduced HbA_1c_ versus placebo, regardless of baseline eGFR (p_INT_ = 0.761) and UACR (p_INT_ = 0.921) (Supplementary Fig. 1). Although semaglutide reduced BW versus placebo in all eGFR subgroups, participants with eGFR ≥ 45–<60 and < 45 mL/min/1.73 m^2^ achieved a greater weight loss than those with eGFR ≥ 60 mL/min/1.73 m^2^ (p_INT_<0.001; Supplementary Fig. 2). Semaglutide consistently reduced BW versus placebo, regardless of baseline UACR (p_INT_ = 0.633).

### Effect of semaglutide on SAEs by baseline kidney function and albuminuria status

The safety profile of semaglutide was also evaluated by the defined eGFR and UACR subgroups. The proportion of participants with SAEs was higher in subgroups with lower eGFR values and higher UACR values at baseline than in the eGFR ≥ 60 mL/min/1.73 m^2^ and UACR < 30 mg/g subgroups, respectively (Supplementary Table 3). The proportion of SAEs was comparable between treatment arms in each subgroup (Supplementary Tables 4 and Supplementary Table 5).

## Discussion

As previously shown in several studies [23–25], this pooled analysis of the SUSTAIN 6 and PIONEER 6 CVOTs validated that the risk of MACE was higher for participants with T2D and at high CV risk with lower eGFR and higher UACR at baseline than for those with eGFR ≥ 60 mL/min/1.73 m^2^ and UACR < 30 mg/g. In line with current guidance [10–12], this study further supports use of semaglutide for the treatment of T2D and CV risk in a broad population with established CVD and a wide range of kidney function and damage. This *post hoc* analysis also showed that there were no additional safety concerns with semaglutide across eGFR and UACR subgroups, supporting and extending the findings for the overall populations in the SUSTAIN 6 and PIONEER 6 CVOTs [5, 6].

The current analysis showed consistent reductions with semaglutide in the risk of MACE versus placebo across baseline eGFR and UACR subgroups, which is in line with a meta-analysis that evaluated CVOTs showing that GLP-1RAs reduced the risk of MACE versus placebo, regardless of baseline eGFR [7]. Results from this *post hoc* analysis and the CVOT meta-analysis are not consistent with observations from the LEADER CVOT [2]. Although the overall risk of MACE was reduced with liraglutide versus placebo in people with T2D and at high CV risk, the relative risk reduction was greater in people with eGFR < 60 mL/min/1.73 m^2^ versus those with ≥ 60 mL/min/1.73 m^2^, indicating that people with reduced kidney function benefitted more from liraglutide treatment in terms of MACE risk [2]. These contrasting results with the current *post hoc* analysis and the CVOT meta-analysis [7] might be attributed to molecular differences between GLP-1RAs, such as semaglutide and liraglutide, affecting the pharmacokinetic and pharmacodynamic properties of each GLP-1RA [1]. Differences in MACE risk with liraglutide versus placebo between eGFR subgroups in the LEADER CVOT [2] might be coincidental or owing to a specific study population. The shorter trial duration of SUSTAIN 6 and PIONEER 6 compared with the 3.5-year trial duration of LEADER might also explain the observed discrepancy in treatment effect across baseline eGFR subgroups [2], because eGFR decline is a continuous process and a follow-up of ≥ 2 years is recommended to reliably predict a treatment effect based on changes in eGFR slope [26].

The current *post hoc* analysis also shows that semaglutide consistently reduced HbA_1c_ versus placebo in people with T2D and a wide range of kidney function. These results confirm previous analyses of the SUSTAIN and PIONEER programmes, showing a consistent reduction in HbA_1c_ across baseline eGFR [17]. However, our results show that participants with eGFR ≥ 45–<60 and < 45 mL/min/1.73 m^2^ achieved a greater weight loss with semaglutide versus placebo than those with eGFR ≥ 60 mL/min/1.73 m^2^, supporting prior evidence [17]. The proportions of gastrointestinal (GI) SAEs were comparable or lower in the low eGFR subgroups compared with the highest eGFR subgroup in our analysis, suggesting that the greater BW reduction observed in the low eGFR subgroups is probably not related to GI SAEs. Importantly, a mediation analysis of the SUSTAIN 3, 7 and 10 trials showed that superior weight loss with OW s.c. semaglutide versus other GLP-1RAs is independent of GI tolerability [27].

In our *post hoc* analysis, the proportions of females were higher in the eGFR ≥ 45–<60 and < 45 mL/min/1.73 m^2^ subgroups than in the eGFR ≥ 60 mL/min/1.73 m^2^ subgroup. This difference in sex distribution at baseline might have contributed to the greater BW reduction in the low eGFR subgroups. Our results are aligned with *post hoc* analyses of the phase 3 STEP programme, evaluating 2.4 mg OW s.c. semaglutide versus placebo in participants with overweight or obesity alone or in addition to T2D, showing that greater weight loss with semaglutide versus placebo is achieved by females than by males [28]. Sex-specific differences in physiology might therefore explain the greater BW reduction in the lower eGFR subgroups than in the high eGFR subgroup. Other minor differences in baseline characteristics across eGFR subgroups, such as the use of antihyperglycaemic medications affecting BW, could potentially contribute to differences in weight loss. Further analyses are warranted to generally understand if the BW reduction is greater in participants with low eGFR in this T2D population at high risk of CVD.

Semaglutide might indirectly improve CV and kidney-related outcomes, but the exact mechanisms by which GLP-1RAs exert beneficial effects on CV and kidney parameters are not yet elucidated, in contrast to the well-established intrarenal tubulo-glomerular feedback mechanism of SGLT2is [13]. However, the known effects of GLP-1RAs on glucose control might contribute to improvements in MACE and kidney-related outcomes. Mediation analyses of the REWIND and LEADER CVOTs showed that reductions in HbA_1c_ and UACR, but not BW, might partly explain a lowered risk of MACE with dulaglutide or liraglutide versus placebo [29, 30]. Although similar *post hoc* analyses of the SUSTAIN 6 CVOT have not indicated that indirect mechanisms play a major role for MACE risk reduction [31, 32], exploratory analyses of the LEADER and SUSTAIN 6 trials showed that 25% and 26%, respectively, of kidney-related benefits are mediated by a glucose-lowering effect of GLP-1RAs; in contrast, no or small mediation by BW was observed [33]. In addition, semaglutide has shown beneficial effects on systolic blood pressure [34] that also have the potential to positively impact CV and kidney-related outcomes.

It is important to note that semaglutide might exert direct mechanisms that can improve CV outcomes; semaglutide treatment altered the intra-cardiac expression of genes related to inflammatory pathways in an atherosclerotic animal model [35]. Furthermore, GLP-1RAs might have direct intrarenal effects, such as reductions in sodium reabsorption, hypoxia, oxidative stress, inflammation and apoptosis, increased natriuresis and haemodynamics, and modified neural and renin–angiotensin–aldosterone system signalling [36, 37]. These direct intrarenal mechanisms of action have not been adequately shown in humans in large-scale trials, but will be addressed in the ongoing REMODEL study [38].

This study provides new insights in the relationship between the multiple effects of semaglutide and baseline CKD status, in addition to providing a rationale for studying kidney-related and CV outcomes in people with T2D and kidney impairment. Kidney-related outcomes will be further evaluated in the FLOW kidney outcomes trial [39] and SOUL CVOT [40] for OW s.c. and OD oral semaglutide, respectively. Because semaglutide reduced the risk of MACE irrespective of baseline kidney function and damage, it is of interest to evaluate if semaglutide can provide consistent effects on hard kidney-related outcomes (kidney failure, ≥ 50% decline in eGFR from baseline, or CV or kidney-related death) in the FLOW trial. In FLOW, the effect of semaglutide on MACE risk will also be evaluated in people with T2D and a wide range of eGFR and UACR at baseline [39, 41]. In SOUL, secondary endpoints evaluating time to first occurrence of a composite of CV and kidney-related events will be studied [40].

The main limitation of this study is the *post hoc* nature of the analyses that may have resulted in random findings. Another limitation is that UACR was not measured in PIONEER 6, leading to fewer number of events included in the analyses. This *post hoc* analysis was not powered for subgroup analyses, and the number of events in certain eGFR and UACR segments was relatively small; this may partly explain the variations between treatment arms observed for ARR and event rate of first MACE across baseline eGFR and UACR values.

## Conclusions

This study shows that MACE risk was higher for participants with reduced kidney function and increased albuminuria at baseline than for those without kidney impairment or damage. The GLP-1RA semaglutide consistently reduced MACE risk across eGFR and UACR subgroups, indicating that semaglutide provides CV benefits in people with T2D and at high CV risk across a broad spectrum of kidney function and damage.

### Electronic supplementary material

Below is the link to the electronic supplementary material.

Supplementary Table 1.pptx. Effect of baseline eGFR and UACR on risk of MACE regardless of treatment. This table shows the unadjusted analysis and adjusted analysis based on a Cox proportional hazards model with adjustment for baseline predictors of cardiorenal disease.

Supplementary Table 2.pptx. Semaglutide vs. placebo on MACE by baseline eGFR and UACR. This table shows the unadjusted analysis and adjusted analysis based on a Cox proportional hazards model with inverse probability weighting, using baseline predictors of cardiorenal disease and continuous eGFR or UACR values at baseline.

Supplementary Table 3.pptx. Participants experiencing serious adverse events by baseline eGFR and UACR. This table shows the numbers and proportions of participants with experience of serious adverse events, categorised by system organ classes, regardless of treatment.

Supplementary Table 4.pptx. Participants experiencing serious adverse events by treatment arm in eGFR subgroups. For each eGFR subgroup, this table shows the numbers and proportions of participants with experience of serious adverse events, categorised by system organ classes, per treatment arm (semaglutide or placebo).

Supplementary Table 5.pptx. Participants experiencing serious adverse events by treatment arm in UACR subgroups. For each UACR subgroup, this table shows the numbers and proportions of participants with experience of serious adverse events, categorised by system organ classes, per treatment arm (semaglutide or placebo).

Supplementary Fig. 1.pptx. The effect of semaglutide on HbA_1c_ by baseline eGFR and UACR. This figure shows the change in HbA_1c_ (%-point) from baseline after treatment with semaglutide or placebo by baseline eGFR or UACR subgroups. Estimated treatment differences, 95% confidence intervals and interaction p values are shown.

Supplementary Fig. 2.pptx. The effect of semaglutide on body weight by baseline eGFR and UACR. This figure shows the change in body weight (kg) from baseline after treatment with semaglutide or placebo by baseline eGFR or UACR subgroups. Estimated treatment differences, 95% confidence intervals and interaction p values are shown.

## Data Availability

The datasets used and/or analysed in the *post hoc* analysis are available from the corresponding author on reasonable request.

## List of abbreviations

ADA: American Diabetes Association
ARR: Absolute risk reduction
BW: Body weight
CI: Confidence interval
CKD: Chronic kidney disease
CV: Cardiovascular
CVD: Cardiovascular disease
CVOT: Cardiovascular outcomes trial
EASD: European Association for the Study of Diabetes
eGFR: Estimated glomerular filtration rate
GI: Gastrointestinal
GLP-1RA: Glucagon-like peptide-1 receptor agonist
HbA_1c_: Glycated haemoglobin
HR: Hazard ratio
KDIGO: Kidney Disease:Improving Global Outcomes
*ln*: Natural logarithm
MACE: Major adverse cardiovascular event
OD: Once-daily
OW: Once-weekly
P1: First percentile
P99: 99th percentile
p_INT_: Interaction p value
Q1: First quartile
Q3: Third quartile
SAE: Serious adverse event
s.c.: Subcutaneous
SGLT2i: Sodium–glucose cotransporter-2 inhibitor
T2D: Type 2 diabetes
UACR: Urine albumin:creatinine ratio
