# Supplementary material for: Effect of semaglutide on major adverse cardiovascular events by baseline kidney parameters in participants with type 2 diabetes and at high risk of cardiovascular disease: SUSTAIN 6 and PIONEER 6 post hoc pooled analysis
Source: Cardiovasc Diabetol. 2023 Aug 24;22:220. doi: 10.1186/s12933-023-01949-7 (PMC10463803; doi:10.1186/s12933-023-01949-7)
Supplement: Supplementary file 1 — Supplementary Table 1.pptx. Effect of baseline eGFR and UACR on risk of MACE regardless of treatment. This table shows the unadjusted analysis and adjusted analysis based on a Cox proportional hazards model with adjustment for baseline predictors of cardiorenal disease. [file 12933_2023_1949_MOESM1_ESM.pptx]

## Slide 1
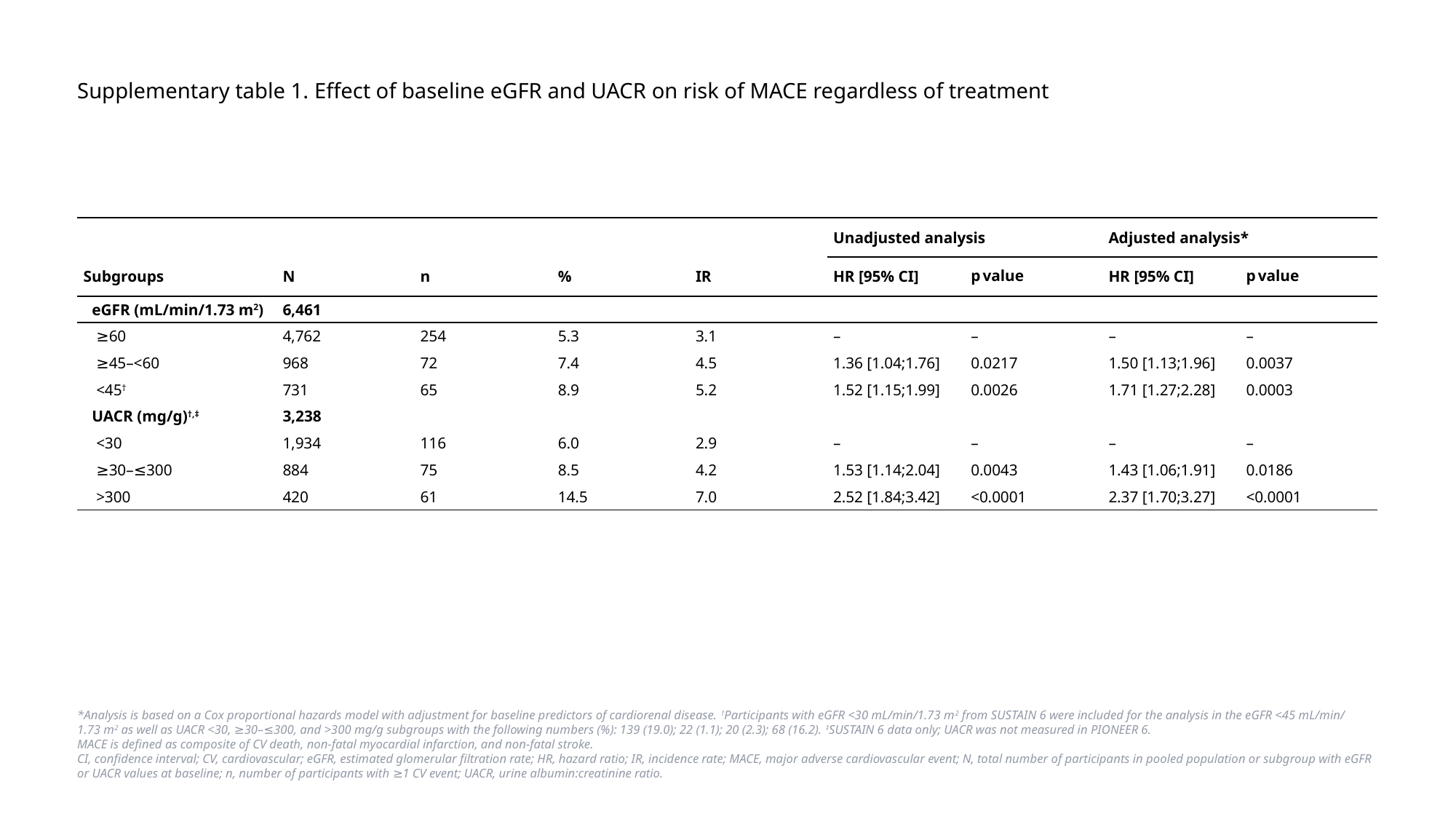

# Supplementary table 1. Effect of baseline eGFR and UACR on risk of MACE regardless of treatment
| | | | | | Unadjusted analysis | | Adjusted analysis\* | |
| --- | --- | --- | --- | --- | --- | --- | --- | --- |
| Subgroups | N | n | % | IR | HR [95% CI] | p value | HR [95% CI] | p value |
| eGFR (mL/min/1.73 m2) | 6,461 | | | | | | | |
| ≥60 | 4,762 | 254 | 5.3 | 3.1 | – | – | – | – |
| ≥45–<60 | 968 | 72 | 7.4 | 4.5 | 1.36 [1.04;1.76] | 0.0217 | 1.50 [1.13;1.96] | 0.0037 |
| <45† | 731 | 65 | 8.9 | 5.2 | 1.52 [1.15;1.99] | 0.0026 | 1.71 [1.27;2.28] | 0.0003 |
| UACR (mg/g)†,‡ | 3,238 | | | | | | | |
| <30 | 1,934 | 116 | 6.0 | 2.9 | – | – | – | – |
| ≥30–≤300 | 884 | 75 | 8.5 | 4.2 | 1.53 [1.14;2.04] | 0.0043 | 1.43 [1.06;1.91] | 0.0186 |
| >300 | 420 | 61 | 14.5 | 7.0 | 2.52 [1.84;3.42] | <0.0001 | 2.37 [1.70;3.27] | <0.0001 |
*Analysis is based on a Cox proportional hazards model with adjustment for baseline predictors of cardiorenal disease. †Participants with eGFR <30 mL/min/1.73 m2 from SUSTAIN 6 were included for the analysis in the eGFR <45 mL/min/
1.73 m2 as well as UACR <30, ≥30–≤300, and >300 mg/g subgroups with the following numbers (%): 139 (19.0); 22 (1.1); 20 (2.3); 68 (16.2). ‡SUSTAIN 6 data only; UACR was not measured in PIONEER 6.
MACE is defined as composite of CV death, non-fatal myocardial infarction, and non-fatal stroke.
CI, confidence interval; CV, cardiovascular; eGFR, estimated glomerular filtration rate; HR, hazard ratio; IR, incidence rate; MACE, major adverse cardiovascular event; N, total number of participants in pooled population or subgroup with eGFR or UACR values at baseline; n, number of participants with ≥1 CV event; UACR, urine albumin:creatinine ratio.
