# Supplementary material for: Effect of semaglutide on major adverse cardiovascular events by baseline kidney parameters in participants with type 2 diabetes and at high risk of cardiovascular disease: SUSTAIN 6 and PIONEER 6 post hoc pooled analysis
Source: Cardiovasc Diabetol. 2023 Aug 24;22:220. doi: 10.1186/s12933-023-01949-7 (PMC10463803; doi:10.1186/s12933-023-01949-7)
Supplement: Supplementary file 2 — Supplementary Table 2.pptx. Semaglutide vs. placebo on MACE by baseline eGFR and UACR. This table shows the unadjusted analysis and adjusted analysis based on a Cox proportional hazards model with inverse probability weighting, using baseline predictors of cardiorenal disease and continuous eGFR or UACR values at baseline. [file 12933_2023_1949_MOESM2_ESM.pptx]

## Slide 1
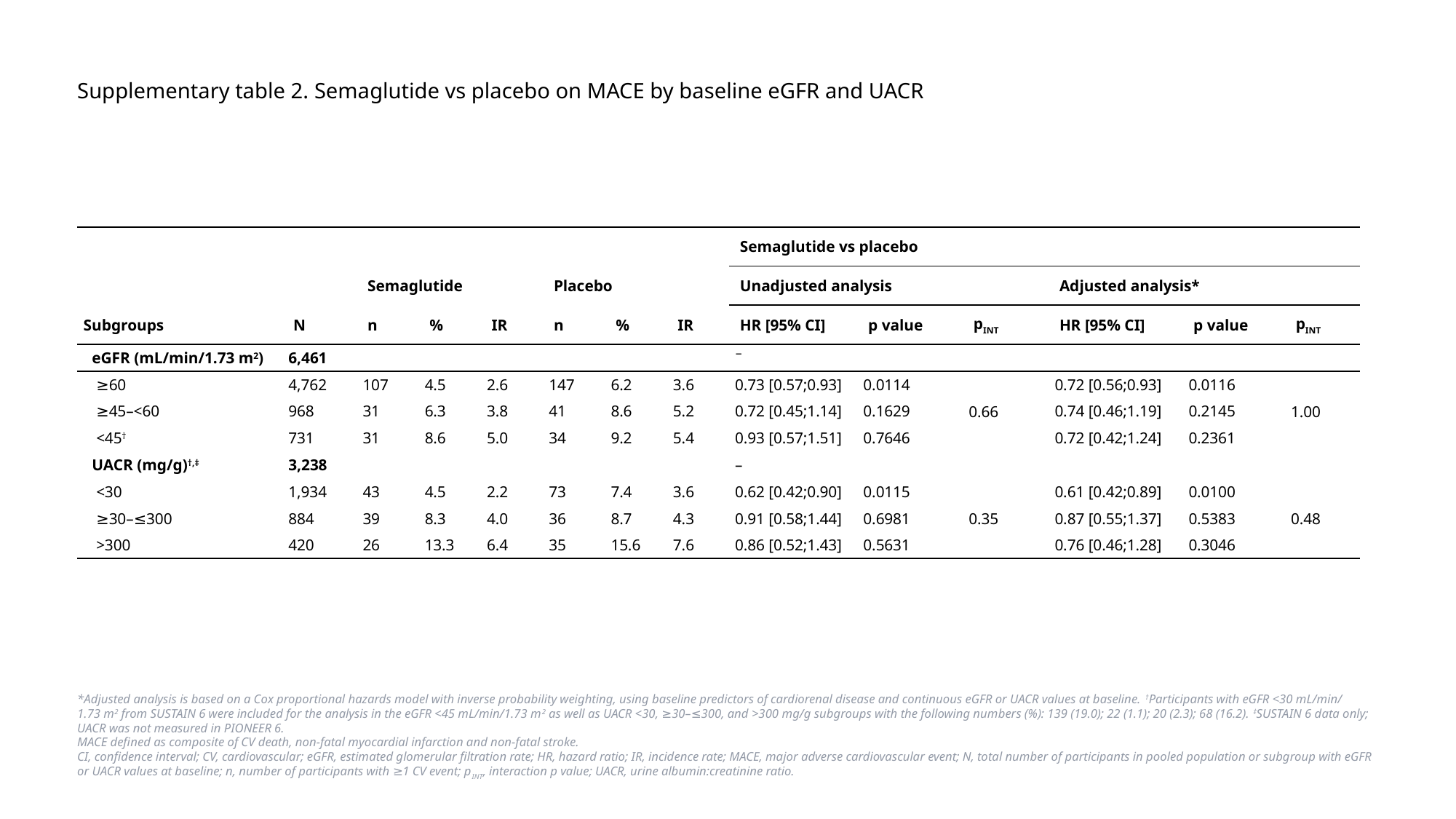

# Supplementary table 2. Semaglutide vs placebo on MACE by baseline eGFR and UACR
| | | | | | | | | Semaglutide vs placebo | | | | | |
| --- | --- | --- | --- | --- | --- | --- | --- | --- | --- | --- | --- | --- | --- |
| | | Semaglutide | | | Placebo | | | Unadjusted analysis | | | Adjusted analysis\* | | |
| Subgroups | N | n | % | IR | n | % | IR | HR [95% CI] | p value | pINT | HR [95% CI] | p value | pINT |
| eGFR (mL/min/1.73 m2) | 6,461 | | | | | | | – | | | | | |
| ≥60 | 4,762 | 107 | 4.5 | 2.6 | 147 | 6.2 | 3.6 | 0.73 [0.57;0.93] | 0.0114 | 0.66 | 0.72 [0.56;0.93] | 0.0116 | 1.00 |
| ≥45–<60 | 968 | 31 | 6.3 | 3.8 | 41 | 8.6 | 5.2 | 0.72 [0.45;1.14] | 0.1629 | | 0.74 [0.46;1.19] | 0.2145 | |
| <45† | 731 | 31 | 8.6 | 5.0 | 34 | 9.2 | 5.4 | 0.93 [0.57;1.51] | 0.7646 | | 0.72 [0.42;1.24] | 0.2361 | |
| UACR (mg/g)†,‡ | 3,238 | | | | | | | – | | | | | |
| <30 | 1,934 | 43 | 4.5 | 2.2 | 73 | 7.4 | 3.6 | 0.62 [0.42;0.90] | 0.0115 | 0.35 | 0.61 [0.42;0.89] | 0.0100 | 0.48 |
| ≥30–≤300 | 884 | 39 | 8.3 | 4.0 | 36 | 8.7 | 4.3 | 0.91 [0.58;1.44] | 0.6981 | | 0.87 [0.55;1.37] | 0.5383 | |
| >300 | 420 | 26 | 13.3 | 6.4 | 35 | 15.6 | 7.6 | 0.86 [0.52;1.43] | 0.5631 | | 0.76 [0.46;1.28] | 0.3046 | |
*Adjusted analysis is based on a Cox proportional hazards model with inverse probability weighting, using baseline predictors of cardiorenal disease and continuous eGFR or UACR values at baseline. †Participants with eGFR <30 mL/min/
1.73 m2 from SUSTAIN 6 were included for the analysis in the eGFR <45 mL/min/1.73 m2 as well as UACR <30, ≥30–≤300, and >300 mg/g subgroups with the following numbers (%): 139 (19.0); 22 (1.1); 20 (2.3); 68 (16.2). ‡SUSTAIN 6 data only; UACR was not measured in PIONEER 6.
MACE defined as composite of CV death, non-fatal myocardial infarction and non-fatal stroke.
CI, confidence interval; CV, cardiovascular; eGFR, estimated glomerular filtration rate; HR, hazard ratio; IR, incidence rate; MACE, major adverse cardiovascular event; N, total number of participants in pooled population or subgroup with eGFR or UACR values at baseline; n, number of participants with ≥1 CV event; pINT, interaction p value; UACR, urine albumin:creatinine ratio.
