# Supplementary material for: Effect of semaglutide on major adverse cardiovascular events by baseline kidney parameters in participants with type 2 diabetes and at high risk of cardiovascular disease: SUSTAIN 6 and PIONEER 6 post hoc pooled analysis
Source: Cardiovasc Diabetol. 2023 Aug 24;22:220. doi: 10.1186/s12933-023-01949-7 (PMC10463803; doi:10.1186/s12933-023-01949-7)
Supplement: Supplementary file 5 — Supplementary Table 5.pptx. Participants experiencing serious adverse events by treatment arm in UACR subgroups. For each UACR subgroup, this table shows the numbers and proportions of participants with experience of serious adverse events, categorised by system organ classes, per treatment arm (semaglutide or placebo). [file 12933_2023_1949_MOESM5_ESM.pptx]

## Slide 1
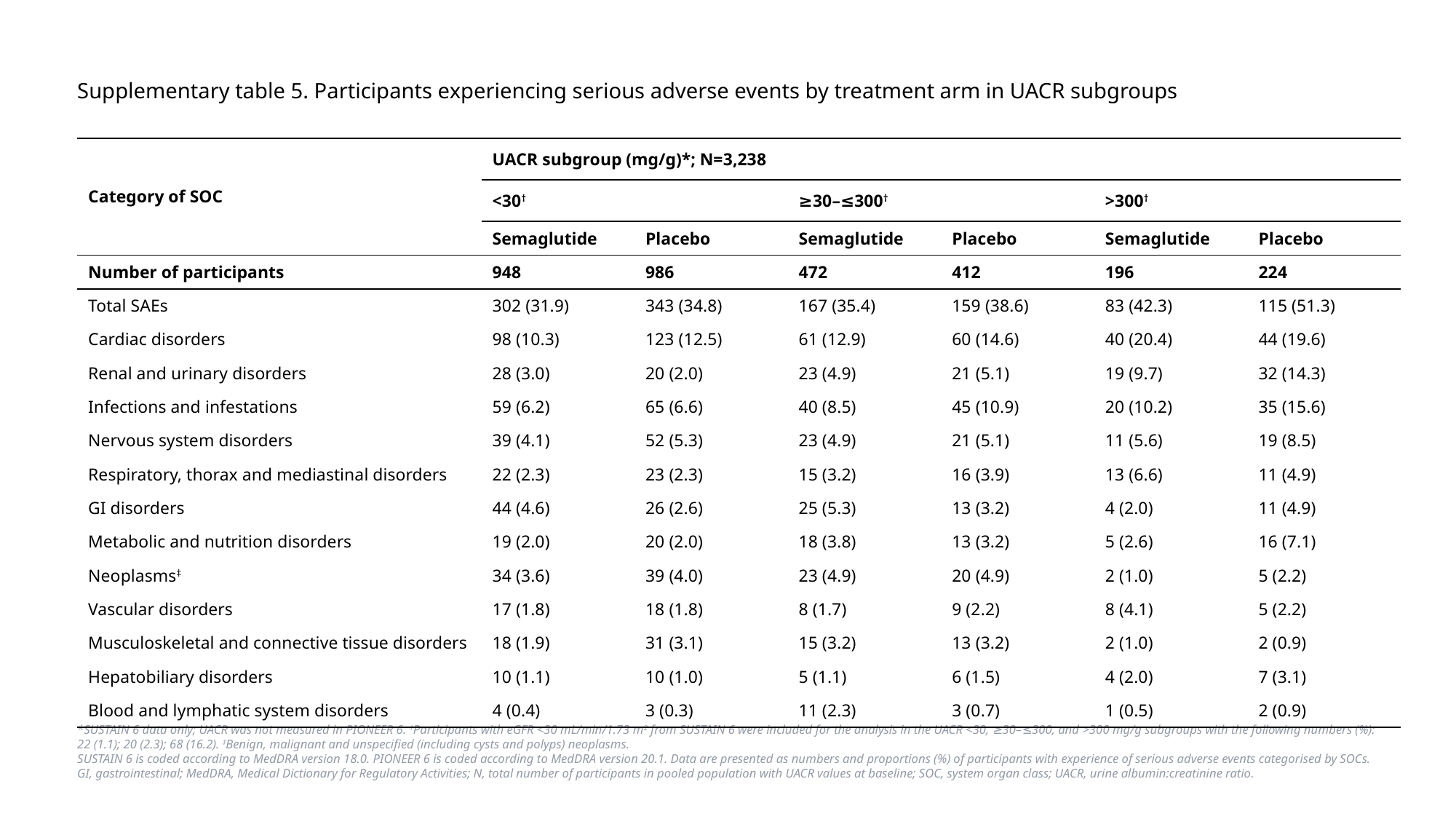

# Supplementary table 5. Participants experiencing serious adverse events by treatment arm in UACR subgroups
| Category of SOC | UACR subgroup (mg/g)\*; N=3,238 | | eGFR subgroup (mL/min/1.73m2) | | | |
| --- | --- | --- | --- | --- | --- | --- |
| | <30† | | ≥30–≤300† | | >300† | |
| | Semaglutide | Placebo | Semaglutide | Placebo | Semaglutide | Placebo |
| Number of participants | 948 | 986 | 472 | 412 | 196 | 224 |
| Total SAEs | 302 (31.9) | 343 (34.8) | 167 (35.4) | 159 (38.6) | 83 (42.3) | 115 (51.3) |
| Cardiac disorders | 98 (10.3) | 123 (12.5) | 61 (12.9) | 60 (14.6) | 40 (20.4) | 44 (19.6) |
| Renal and urinary disorders | 28 (3.0) | 20 (2.0) | 23 (4.9) | 21 (5.1) | 19 (9.7) | 32 (14.3) |
| Infections and infestations | 59 (6.2) | 65 (6.6) | 40 (8.5) | 45 (10.9) | 20 (10.2) | 35 (15.6) |
| Nervous system disorders | 39 (4.1) | 52 (5.3) | 23 (4.9) | 21 (5.1) | 11 (5.6) | 19 (8.5) |
| Respiratory, thorax and mediastinal disorders | 22 (2.3) | 23 (2.3) | 15 (3.2) | 16 (3.9) | 13 (6.6) | 11 (4.9) |
| GI disorders | 44 (4.6) | 26 (2.6) | 25 (5.3) | 13 (3.2) | 4 (2.0) | 11 (4.9) |
| Metabolic and nutrition disorders | 19 (2.0) | 20 (2.0) | 18 (3.8) | 13 (3.2) | 5 (2.6) | 16 (7.1) |
| Neoplasms‡ | 34 (3.6) | 39 (4.0) | 23 (4.9) | 20 (4.9) | 2 (1.0) | 5 (2.2) |
| Vascular disorders | 17 (1.8) | 18 (1.8) | 8 (1.7) | 9 (2.2) | 8 (4.1) | 5 (2.2) |
| Musculoskeletal and connective tissue disorders | 18 (1.9) | 31 (3.1) | 15 (3.2) | 13 (3.2) | 2 (1.0) | 2 (0.9) |
| Hepatobiliary disorders | 10 (1.1) | 10 (1.0) | 5 (1.1) | 6 (1.5) | 4 (2.0) | 7 (3.1) |
| Blood and lymphatic system disorders | 4 (0.4) | 3 (0.3) | 11 (2.3) | 3 (0.7) | 1 (0.5) | 2 (0.9) |
*SUSTAIN 6 data only; UACR was not measured in PIONEER 6. †Participants with eGFR <30 mL/min/1.73 m2 from SUSTAIN 6 were included for the analysis in the UACR <30, ≥30–≤300, and >300 mg/g subgroups with the following numbers (%):
22 (1.1); 20 (2.3); 68 (16.2). ‡Benign, malignant and unspecified (including cysts and polyps) neoplasms.
SUSTAIN 6 is coded according to MedDRA version 18.0. PIONEER 6 is coded according to MedDRA version 20.1. Data are presented as numbers and proportions (%) of participants with experience of serious adverse events categorised by SOCs.
GI, gastrointestinal; MedDRA, Medical Dictionary for Regulatory Activities; N, total number of participants in pooled population with UACR values at baseline; SOC, system organ class; UACR, urine albumin:creatinine ratio.
