# Supplementary material for: Effect of semaglutide on major adverse cardiovascular events by baseline kidney parameters in participants with type 2 diabetes and at high risk of cardiovascular disease: SUSTAIN 6 and PIONEER 6 post hoc pooled analysis
Source: Cardiovasc Diabetol. 2023 Aug 24;22:220. doi: 10.1186/s12933-023-01949-7 (PMC10463803; doi:10.1186/s12933-023-01949-7)
Supplement: Supplementary file 6 — Supplementary Fig. 1.pptx. The effect of semaglutide on HbA1c by baseline eGFR and UACR. This figure shows the change in HbA1c (%-point) from baseline after treatment with semaglutide or placebo by baseline eGFR or UACR subgroups. Estimated treatment differences, 95% confidence intervals and interaction p values are shown. [file 12933_2023_1949_MOESM6_ESM.pptx]

## Slide 1
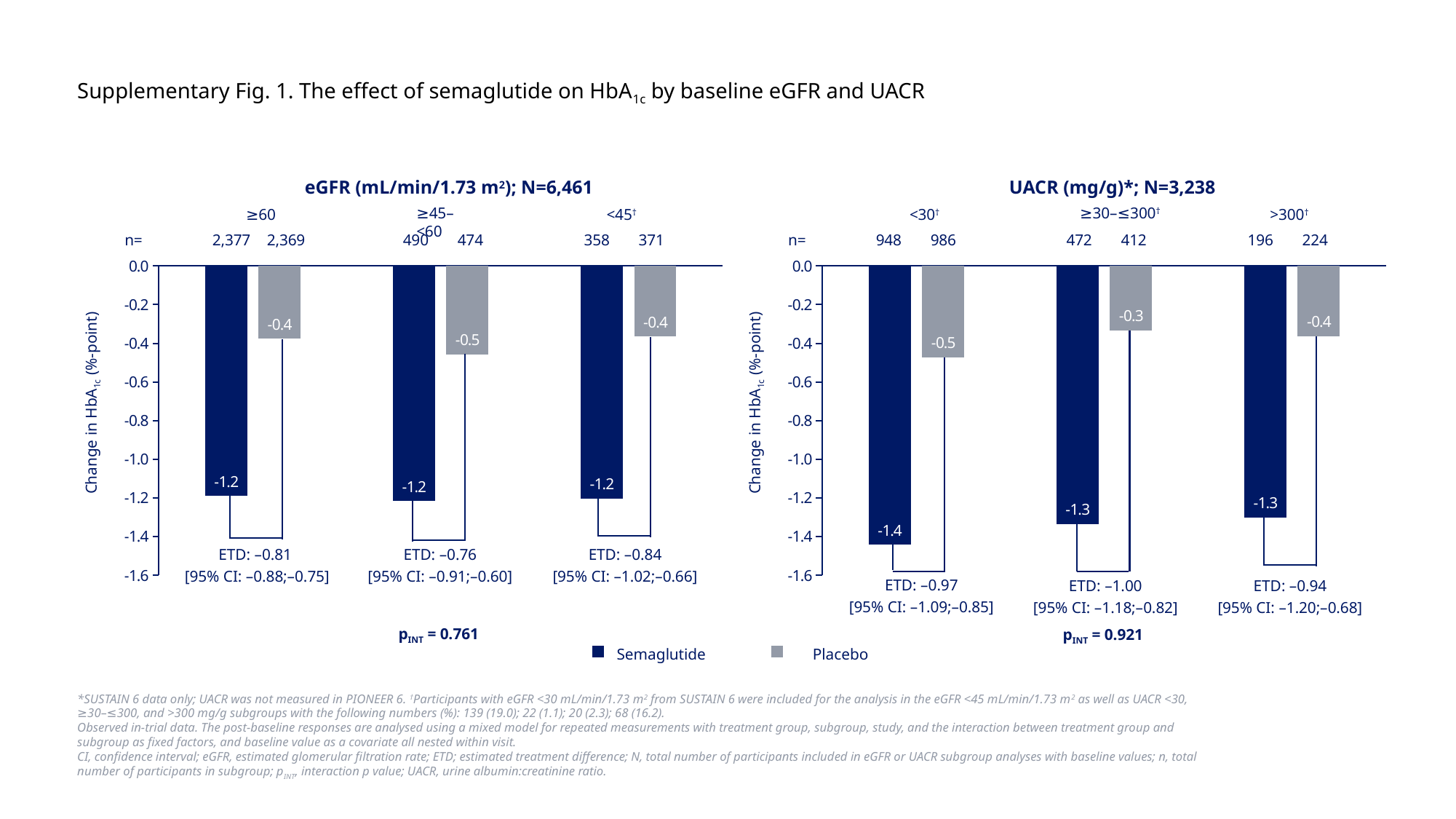

# Supplementary Fig. 1. The effect of semaglutide on HbA1c by baseline eGFR and UACR
eGFR (mL/min/1.73 m2); N=6,461
UACR (mg/g)*; N=3,238
≥45–<60
≥30–≤300†
≥60
<45†
<30†
>300†
490
474
472
412
358
371
196
224
n=
2,377
2,369
n=
948
986
### Chart
| Category | Semaglutide | Placebo |
|---|---|---|
| >60 | -1.19 | -0.376 |
| >45-<60 | -1.216 | -0.458 |
| <45 | -1.202 | -0.364 |Change in HbA1c (%-point)
### Chart
| Category | Semaglutide | Placebo |
|---|---|---|
| >30 | -1.442 | -0.472 |
| >30-<300 | -1.334 | -0.333 |
| >300 | -1.3 | -0.362 |Change in HbA1c (%-point)
ETD: –0.81
[95% CI: –0.88;–0.75]
ETD: –0.76
[95% CI: –0.91;–0.60]
ETD: –0.84
[95% CI: –1.02;–0.66]
ETD: –0.97
[95% CI: –1.09;–0.85]
ETD: –0.94
[95% CI: –1.20;–0.68]
ETD: –1.00
[95% CI: –1.18;–0.82]
pINT = 0.761
pINT = 0.921
Semaglutide
Placebo
*SUSTAIN 6 data only; UACR was not measured in PIONEER 6. †Participants with eGFR <30 mL/min/1.73 m2 from SUSTAIN 6 were included for the analysis in the eGFR <45 mL/min/1.73 m2 as well as UACR <30,
≥30–≤300, and >300 mg/g subgroups with the following numbers (%): 139 (19.0); 22 (1.1); 20 (2.3); 68 (16.2).
Observed in-trial data. The post-baseline responses are analysed using a mixed model for repeated measurements with treatment group, subgroup, study, and the interaction between treatment group and subgroup as fixed factors, and baseline value as a covariate all nested within visit.
CI, confidence interval; eGFR, estimated glomerular filtration rate; ETD; estimated treatment difference; N, total number of participants included in eGFR or UACR subgroup analyses with baseline values; n, total number of participants in subgroup; pINT, interaction p value; UACR, urine albumin:creatinine ratio.
