# Supplementary material for: Effect of semaglutide on major adverse cardiovascular events by baseline kidney parameters in participants with type 2 diabetes and at high risk of cardiovascular disease: SUSTAIN 6 and PIONEER 6 post hoc pooled analysis
Source: Cardiovasc Diabetol. 2023 Aug 24;22:220. doi: 10.1186/s12933-023-01949-7 (PMC10463803; doi:10.1186/s12933-023-01949-7)
Supplement: Supplementary file 7 — Supplementary Fig. 2.pptx. The effect of semaglutide on body weight by baseline eGFR and UACR. This figure shows the change in body weight (kg) from baseline after treatment with semaglutide or placebo by baseline eGFR or UACR subgroups. Estimated treatment differences, 95% confidence intervals and interaction p values are shown. [file 12933_2023_1949_MOESM7_ESM.pptx]

## Slide 1
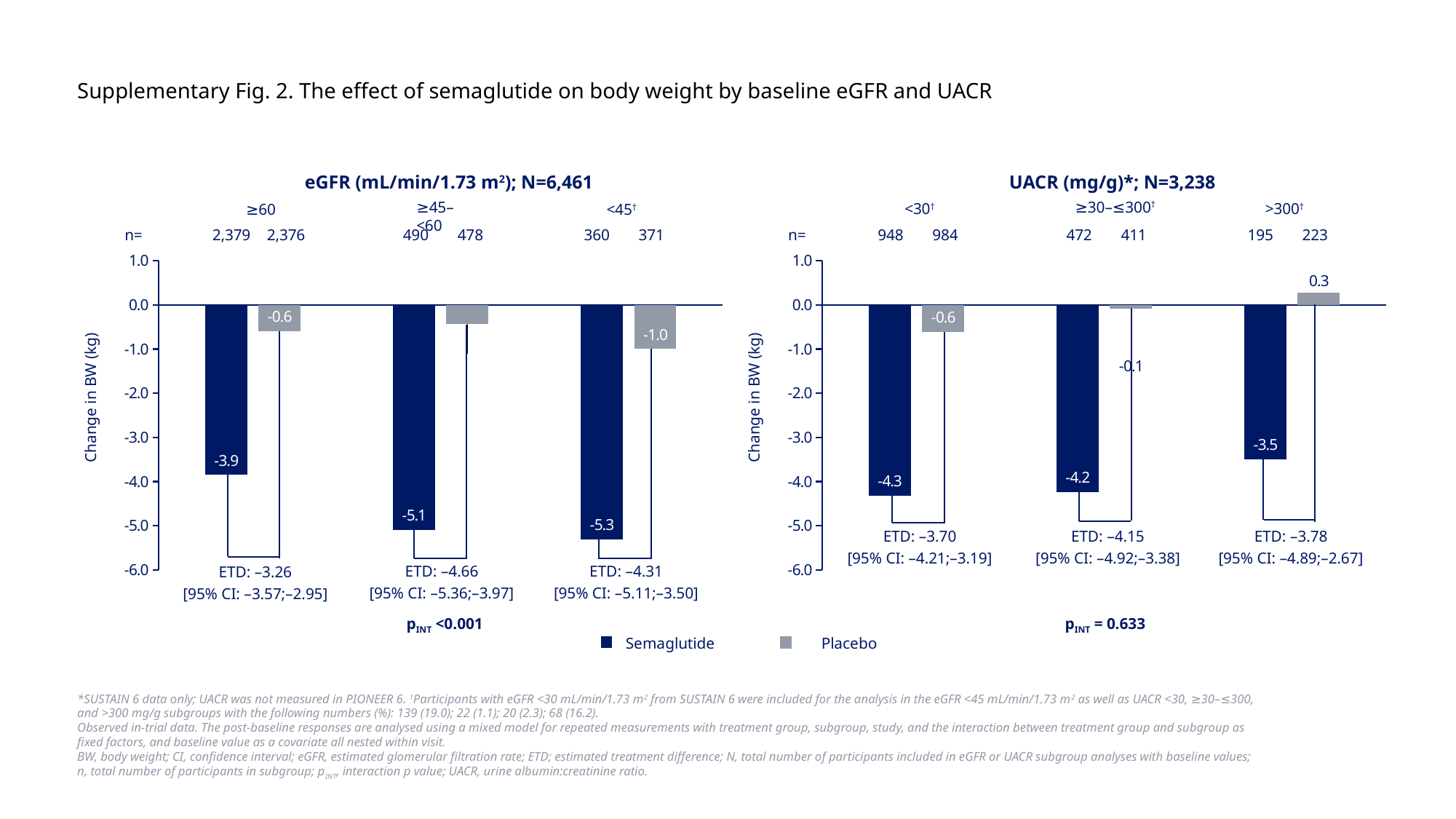

# Supplementary Fig. 2. The effect of semaglutide on body weight by baseline eGFR and UACR
eGFR (mL/min/1.73 m2); N=6,461
UACR (mg/g)*; N=3,238
≥30–≤300†
≥45–<60
<30†
>300†
≥60
<45†
490
478
948
984
472
411
360
371
195
223
n=
2,379
2,376
n=
### Chart
| Category | Semaglutide | Placebo |
|---|---|---|
| >60 | -3.851 | -0.59 |
| >45-<60 | -5.094 | -0.433 |
| <45 | -5.307 | -0.998 |Change in BW (kg)
### Chart
| Category | Semaglutide | Placebo |
|---|---|---|
| >30 | -4.315 | -0.615 |
| >30-<300 | -4.232 | -0.084 |
| >300 | -3.499 | 0.279 |Change in BW (kg)
ETD: –3.78
[95% CI: –4.89;–2.67]
ETD: –4.15
[95% CI: –4.92;–3.38]
ETD: –3.70
[95% CI: –4.21;–3.19]
ETD: –4.66
[95% CI: –5.36;–3.97]
ETD: –4.31
[95% CI: –5.11;–3.50]
ETD: –3.26
[95% CI: –3.57;–2.95]
pINT <0.001
pINT = 0.633
Semaglutide
Placebo
*SUSTAIN 6 data only; UACR was not measured in PIONEER 6. †Participants with eGFR <30 mL/min/1.73 m2 from SUSTAIN 6 were included for the analysis in the eGFR <45 mL/min/1.73 m2 as well as UACR <30, ≥30–≤300, and >300 mg/g subgroups with the following numbers (%): 139 (19.0); 22 (1.1); 20 (2.3); 68 (16.2).
Observed in-trial data. The post-baseline responses are analysed using a mixed model for repeated measurements with treatment group, subgroup, study, and the interaction between treatment group and subgroup as fixed factors, and baseline value as a covariate all nested within visit.
BW, body weight; CI, confidence interval; eGFR, estimated glomerular filtration rate; ETD; estimated treatment difference; N, total number of participants included in eGFR or UACR subgroup analyses with baseline values;
n, total number of participants in subgroup; pINT, interaction p value; UACR, urine albumin:creatinine ratio.
